# Expression of miRNAs from the Imprinted *DLK1/DIO3* Locus Signals the Osteogenic Potential of Human Pluripotent Stem Cells

**DOI:** 10.3390/cells8121523

**Published:** 2019-11-26

**Authors:** Laetitia Barrault, Jacqueline Gide, Tingting Qing, Lea Lesueur, Jorg Tost, Jerome Alexandre Denis, Michel Cailleret, Laetitia Aubry, Marc Peschanski, Cécile Martinat, Sandrine Baghdoyan

**Affiliations:** 1 CECS/AFM, I-STEM, 91100 Corbeil-Essonnes, France; lbarrault@istem.fr (L.B.); jgide@istem.fr (J.G.); tt.qing@gmail.com (T.Q.); mpeschanski@istem.fr (M.P.); 2 INSERM/ UEVE UMR 861, Paris Saclay Univ I-STEM, 91100 Corbeil-Essonnes, France; llesueur@istem.fr (L.L.); jerome.denis@aphp.fr (J.A.D.); mcailleret@istem.fr (M.C.); laubry@istem.fr (L.A.); sbaghdoyan@istem.fr (S.B.); 3 LEE/ CNRGH/ CEA—IBFJ 2, 91000 Evry, France; jorg.tost@cng.fr

**Keywords:** human pluripotent stem cells, microRNA, imprinting, osteogenic differentiation, variability, ACVR2B

## Abstract

Substantial variations in differentiation properties have been reported among human pluripotent cell lines (hPSC), which could affect their utility and clinical safety. We characterized the variable osteogenic capacity observed between different human pluripotent stem cell lines. By focusing on the miRNA expression profile, we demonstrated that the osteogenic differentiation propensity of human pluripotent stem cell lines could be associated with the methylation status and the expression of miRNAs from the imprinted *DLK1/DIO3* locus. More specifically, quantitative analysis of the expression of six different miRNAs of that locus prospectively identified human embryonic stem cells and human-induced pluripotent stem cells with differential osteogenic differentiation capacities. At the molecular and functional levels, we showed that these miRNAs modulated the expression of the activin receptor type 2B and the downstream signal transduction, which impacted osteogenesis. In conclusion, miRNAs of the imprinted *DLK1/DIO3* locus appear to have both a predictive value and a functional impact in determining the osteogenic fate of human pluripotent stem cells.

## 1. Introduction

From embryonic origin or generated by the direct reprogramming of somatic cells, human pluripotent stem cells (hPSCs) are now starting to live up to the great expectations they created after their first derivation twenty years ago [1]. This was exemplified by the recently published results of cell-based clinical trials to treat macular degeneration [2] and the increasing number of clinical trials launched for broader conditions such as spinal cord injury and heart disease [3,4,5].

The main conditions for these developments rely on the ‘holy grail’ to efficiently and robustly differentiate hPSCs into cell types of interest [6]. However, these conversions are often hindered by the differentiation inconsistency of individual hPSC lines, which consequently result in time-consuming and costly optimization to yield the cell type of interest [7]. This highlights the necessity to effectively and rapidly assess these biological resources with respect to their ability to differentiate into the specific cell type of interest. The expression level of single genes has been shown to be predictive of the differentiation propensity of neural, endodermal, and hematopoietic lineages [8,9,10,11,12,13]. This concept has been extended to noncoding RNAs such as miR-371–3p, which can predict neural differentiation propensity in undifferentiated human pluripotent stem cells and even their potential to generate specifically dopaminergic neurons that engraft in vivo [8].

The identification of predictive markers not only offers a practical strategy to select appropriate lines for translational applications, but also opens the path for exploring the molecular mechanisms associated with cell line-specific differences. In this context, hPSCs have recently been demonstrated to be a promising source of osteoblasts for bone regeneration [14,15,16,17,18,19]. However, the osteogenic behavior among different human pluripotent stem cell lines is still unclear. In this research, we demonstrated a variable capacity of osteogenesis between 10 different hPSC lines. The molecular mechanisms of inter-cell line variability involve differences in the epigenetic control of miRNAs at the *DLK1/DIO3* imprinted locus, which modulates the activin receptor 2B expression and consequently, the osteogenic potential of hPSC lines.

## 2. Materials and Methods

### 2.1. Pluripotent Stem Cell Culture and Mesodermal Differentiation

Human embryonic stem cell (hESC) lines were used following the recommendation of the French Law of Bioethics and declared at the French Agency of Biomedicine (Number SASB1020178S). hESC lines H9 (WA-09), SA01, and VUB03_DM were obtained from WiCell Research Institute, Cellectis/Cellartis, and the Department of Embryology and Genetics of the Vrije Universiteit, AZ-VUB Laboratory, Brussels, Belgium, respectively. The SA01 line overexpressing ACVR2B was generated by stable transfection using Lipofectamie 3000 from the ACVR2B coding sequence inserted by Gibson cloning in the EcoRI enzymatic site of the pAAVS1-P-CAG-DEST vector (pAAVS1-P-CAG-DEST was a gift from Knut Woltjen (Addgene^®^ Ref#80490; http://n2t.net/addgene:80490; RRID: Addgene_80490)). The PC056 and PC060 human-induced pluripotent stem cells (hiPSCs) (Phenocell^®^; Grasse; France) were derived from human primary fibroblasts and were reprogrammed using sendai vectors expressing OCT4, KLF4, SOX2, and c-Myc [20]. The hiPSCs lines 4603, 3814, 1869, I90, and FS2 were reprogrammed using episomal vectors expressing OCT4, SOX2, NANOG, and LIN28 [21] starting from human primary fibroblasts (Coriell GM04603, GM03814, GM01869 and IMR-90) and human foreskin (FS), respectively. Pluripotent stem cell lines were manually dissected and plated on mitotically inactivated embryonic mouse fibroblasts in DMEM/F12 glutamax supplemented with 20% knockout serum replacement, 1 mM nonessential amino acids, 1% penicillin/streptomycin, 0.55 mM 2-mercaptoethanol, and 5 ng/ml recombinant human FGF2 (all from Invitrogen/ Thermofisher Scientific^®^; Villebon sur Yvette; France). Mesodermal differentiation was induced as previously described [22]. Briefly, 2.10^4^ hES cells/cm2 were plated on 0.1% gelatin-coated dishes in the presence of knockout DMEM supplemented with 20% fetal bovine serum, 1 mM l-glutamine, 1% nonessential amino acids, 0.1 mM β-mercaptoethanol, ascorbic acid 2-phosphate 1 mM (Sigma-Aldrich^®^; Saint Quentin; France), and FGF2 10 ng/mL (all from Invitrogen/Thermofischer Scientific^®^). The medium was changed every 3 days.

### 2.2. Surface Antigen Analysis

Cell surface antigens on hiPS and hESC-mesodermal progenitor cells (MPCs) were analyzed using fluorescence-activated cell sorting (FACS). The cells were dissociated into single cells with trypsin, resuspended in 0.1%BSA-PBS, and incubated for 30 min at room temperature with fluorescence-conjugated antibodies. The antibodies used for FACS were mouse antihuman CD29 conjugated with fluorescein isothiocyanate (FITC), mouse antihuman CD105 conjugated with phycoerythrin coupled with cyanin 7 (PE-Cy7), mouse antihuman CD44 conjugated with allophycocyanin coupled with cyanin (APC-Cy7), mouse antihuman CD166 conjugated with phycoerythrin (PE), and mouse antihuman CD73 conjugated with allophycocyanin (APC). All the antibodies were purchased from BD Bioscience. Appropriate antibodies were used as a negative control. The cells were washed twice with 0.1%BSA-PBS and were then suspended in 0.5 mL of 0.1% BSA-PBS for analysis with a Macs Quant (Miltenyi Biotec^®^; Paris; France). More than 10,000 events were acquired for each sample and were analyzed. Data retrieved from the sorting were analyzed with FlowJo software (FlowJo LLC/ Miltenyi Biotec^®^; Paris, France ).

### 2.3. Osteogenic Differentiation

MPCs were washed once with PBS and cultured in a STEMPro Osteogenesis Differentiation Kit (Invitrogen/ Thermofischer Scientific ^®^). Differentiation of the cultures was tested on day 10 for the detection of alkaline phosphatase activity with SIGMAFAST™ BCIP^®^/NBT (Sigma-Aldrich^®^) and alizarin red staining with alizarin red Staining solution (Merck/ Millipore^®^ Saint Quentin; France) on day 20 according the manufacturer’s instructions. Total cell number during differentiation was monitored with the CellTiter-Glo assay (Promega^®^; Charbonnie; France) according to the manufacturer’s instructions.

### 2.4. Mesodermal Progenitor Cell Transfection

MPCs were transfected 24 h after plating at 2.5 × 10^4^ cells/cm^2^ in a 24-well plate in knockout DMEM containing 20% of fetal bovine serum (Eurobio^®^), 1% Glutamax and 1% nonessential amino acids (Invitrogen/ Thermofischer Scientific^®^). For the pre-microRNA overexpression experiments, cells were transfected in OptiMEM medium using Lipofectamine™ RNAiMax reagent (Invitrogen/ Thermofischer Scientific ^®^) with 10 nM of the AllStars Neg. Control siRNA (#1027281) or different miScript miRNA mimic from Qiagen^®^ (Les Ullis; France). References of miScript miRNA mimics are described in Table S1.

### 2.5. HEK293T Cells Transfection

HEK293T cells were plated at 6 × 10^4^ cells/cm^2^ in a 96-well plate in alpha MEM medium supplemented with 10% SVF (Hyclone^®^) and 1% Glutamax, 1% nonessential amino acids, and 1% penicillin–streptomycin. The cells were transfected with 10 nmol/l of the AllStars Neg. Control siRNA (#1027281), siRNA ACVR2B_3, and pre-miR 377-3p, pre-miR-410, and pre-miR-543 (all from Qiagen^®^) in OptiMEM medium using 1 µL LipoRNAiMax (Invitrogen/ Thermofischer Scientific ^®^). For Luciferase assays, the cells were cotransfected twenty-four hours after plating with 10 nM of siRNA ACVR2B_3 (Qiagen^®^) or miScript miRNA miR-377 Mimic (Qiagen^®^) and 100 ng of SBE reporter plasmids (Cignal SMAD Reporter Assay Kit (LUC) (CCS-017L) (Qiagen^®^)) using Lipofectamine™ 2000 Reagent (Invitrogen/ Thermofischer Scientific ^®^). Twenty-four hours after transfection, the activity of the SBE-reporter plasmid was revealed using the Dual-Glo^®^ Luciferase Assay System (Promega^®^). The luminescent signal was quantified on a CLARIOstar plate reader (BMG Labtech^®^; Champigny sur Marne, France).

### 2.6. Methylation Assays by Pyrosequencing

Genomic DNA (1 μg) was treated with sodium bisulfite using EpiTect^®^ 96 Bisulfite (Qiagen^®^) according to the manufacturer’s instructions. Quantitative DNA methylation analysis of the bisulphite-treated DNA was performed by pyrosequencing, or, in the case of several sequencing primers, by serial pyrosequencing on a PSQ 96MD system with the PyroGold SQA reagent kit (Biotage AB^®^), and results were analyzed using Q-CpG software (V.1.0.9, Biotage^®^; Uppsala, Sweden) as previously described [23].

### 2.7. Protein Extraction and Western Blot Analysis

Cells were homogenized in radioimmunoprecipitation assay buffer (Sigma-Aldrich^®^) containing 1% protease inhibitors and 10% phosphatase inhibitors (Sigma-Aldrich^®^). After electrophoresis on 4%–12% Nu-PAGE Bis-Tris gels (Invitrogen^®^) under reducing conditions, proteins were transferred to nitrocellulose membranes (Invitrogen^®^), blocked with Odissey blocking buffer containing 0.1% Tween-20, and incubated overnight with the primary antibody diluted in the Odissey blocking buffer containing 0.1% Tween-20. Membranes were then incubated for 1 h with the corresponding IRDye secondary antibodies (LI-COR^®^; Bad Homburg, Germany) and immunoreactive protein bands were detected using an Odissey CLx Imager (LI-COR) according to the manufacturer’s protocol. Antibodies used in Western blotting analysis were anti-activin receptor type IIB antibody-ab76940 1/1000 (Abcam), anti-ACVR1 antibody-ab155981 1/1000, and anti-ACTB antibody-92642210 1/15000 (LI-COR^®^).

### 2.8. Gene Expression Analysis

Total RNA was extracted using the RNeasy Micro/Mini kit (Qiagen^®^) and was reverse transcribed using random hexamers and the Superscript III reverse transcriptase kit (Invitrogen/ Thermofischer Scientific^®^). Quantitative PCR reactions were carried out in 384-well plates using a QuantStudio 12K Flex Real-Time PCR System (Applied Biosystems^®^) with Power SYBR Green 2× Master Mix (Life Technologies/ Thermofischer Scientific^®^), 0.5 μl of cDNA, and 100 nmol/l of primers (Invitrogen/ Thermofischer Scientific ^®^) in a final volume of 10 μL. The relative expression level of each gene was calculated with the method described by Pfaffl [24]. Primer sequences are described in Table S1.

TGFβ/BMP Signaling Pathway Plus PCR Array PAHS-035 (Qiagen^®^) was used to monitor the expression of genes belonging to the transforming growth factor-β (TGFβ)/BMP (Bone morphogenetic protein) signaling pathway according the manufacturer’s instructions.

### 2.9. MicroRNA Expression Analysis

MicroRNAs were extracted using the NucleoSpin miRNA kit (Macherey Nagel^®^) and were reverse-transcribed using the miScript II RT kit (Qiagen^®^). Quantitative PCR reactions were carried out in 384-well plates using a QuantStudio 12K Flex Real-Time PCR System (Applied Biosystems/ Thermofischer Scientific^®^) with the miScript SYBR Green PCR kit (Qiagen^®^) and the assays from Qiagen described in Supplemental Table S1. The HsRNU6-21 miScript Primer Assay was used for the normalization of microRNA expression. Analysis of the expression profiling of 754 microRNAs in hES cell lines and MPCS was carried out on three replicates for each RNA sample according to the recommendation of Applied Biosystems. The assay included RT with specific primers, followed by real-time qPCR using the TaqMan Array Human microRNA A + B Cards set v3.0 and TaqMan universal master mix in an Applied Biosystems 7900 Real-Time PCR System. MicroRNA expression levels were normalized to two different internal control small RNAs (RNU48 and U6 snRNA), obtaining similar results. The comparative threshold cycle method was used to calculate the relative microRNA expression.

### 2.10. Target Gene Prediction

The TargetScanHuman database version 7.1 [25] was used to predict genes targeted by miRNA from the *DLK1/DIO3* locus. The gene list was then analyzed using the Panther classification system to identify the enriched pathways among the identified putative microRNA targets.

### 2.11. Statistical Analysis

Statistics were computed with GraphPad Prism 5.0 software (GraphPad Software, Inc; San Diego, US.). Values are reported as the mean and SD or the mean and SEM according to the experiments. Differences between groups were considered significant when *p* < 0.05 (* *p* < 0.05; ** *p* < 0.01; *** *p* < 0.001). According to the size of the experiment, parametric or nonparametric tests were chosen (*t* test, one-way or two-way ANOVA, and post hoc tests).

## 3. Results

### 3.1. Human Pluripotent Stem Cells Differentiate between Mesenchymal Progenitors with Variable Osteogenic Capacity

To evaluate the potential of different human pluripotent stem cell lines (hPSCs) to differentiate between osteogenic lineage, three different human embryonic stem cell lines (hESCs) (VUB03, SA01, and H9) and three different human-induced pluripotent stem cells (hiPSCs) (4603, 3814, and FS2) were sequentially converted into mesodermal progenitor cells, followed by osteoblast induction and maturation phases (Figure S1). As previously described by our group, we first confirmed that these six hPSC lines were all present in the same capacity in to differentiate into a homogeneous population of mesodermal progenitor cells (PSC_MPCs) (Figure S1) [22]. Indeed, flow cytometry analysis revealed that more than 95% of hPSC_MPCs expressed CD73, CD29, CD105, CD44, and CD166, key stromal surface markers.

**Figure 1:**
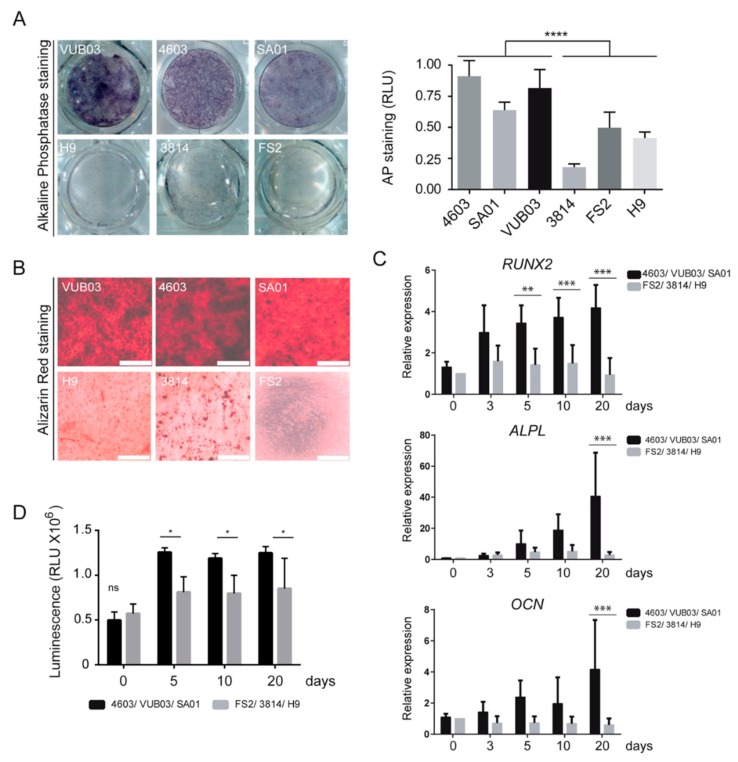
Heterogeneity in osteogenic differentiation potential of mesodermal progenitor cells (MPCs) derived from human pluripotent stem cell (hPSC-MPC) lines. (**A**) Comparison and quantification of alkaline phosphatase staining in MPCs derived from six hPSC lines, VUB03, 4603, SA01, H9, 3814, and FS2, cultured for ten days in osteogenic differentiation medium. Data obtained from 3 independent experiments are represented as the mean ± SD. Statistical analysis was based on an unpaired *t*-Test; **** *p* < 0.0001. (**B**) Comparison of alizarin red staining in MPCs derived from six hPSC lines, cultured for twenty days in osteogenic differentiation medium. Data correspond to 3 independent experiments (Scale bar: 500 µM). (**C**) Time-course analysis of *RUNX2*, *ALPL,* and *OCN* gene expression by quantitative RT-QPCR analysis in VUB03, 4603, and SA01 MPCs versus H9, 3814, and FS2 MPCs. Data obtained from independent experiments for the 6 hPSC lines are represented as the mean ± SD. (**D**) Time-course analysis of viable cell number evaluated by the ATP content during osteogenic differentiation of MPCs VUB03, 4603, SA01 compared to MPCs H9, 3814, FS2 with poor differentiation potential. Data obtained from 3 independent quantifications for each cell line are represented as the mean ± SD. For C and D, statistical analysis was based on a two-way ANOVA followed by a Sidak’s multiple comparison. * *p* < 0.05, ** *p* < 0.01, *** *p* < 0.001, ns: not significant.

The differentiation of these hPSC_MPCs into osteogenic lineage was evaluated by measuring alkaline phosphatase activity and mineralization assay after 10 and 20 days, respectively, in an osteogenic differentiation medium. Strikingly, we observed a differential capacity of these six different lines of hPSC_MPCs to differentiate into the osteogenic lineage: whereas VUB03, 4603, and SA01_MPCs harbored a robust level of alkaline phosphatase activity and calcium deposition after osteogenic differentiation, H9, 3814, and FS2_MPCs remained almost negative for these differentiation markers (Figure 1A,B). This phenotype was confirmed at the molecular level by quantifying the expression of osteogenic markers, such as runt-related transcription factor 2 (*RUNX2*), alkaline phosphatase (*ALPL*), and osteocalcin (*OCN*), by RT-qPCR analysis on days 0, 3, 5, 10, and 20 after the initiation of differentiation. Transcript levels of *RUNX2* and *ALPL* remained lower in FS2, 3814, and H9_MPCs compared to 4603, VUB03, and SA01_MPCs, while no induction of *OCN* was detected in these cells on day 20 (Figure 1C). This defective differentiation was coupled with a reduced number of cells on days 10 and 20 of differentiation in FS2, 3814, and H9_MPCs based on quantitation of the Adenosine TriPhosphate (ATP) present in metabolically active cells (Figure 1D).

### 3.2. The Osteogenic Potential of Human Pluripotent Stem Cells Is Correlated with the Expression of DLK1/DIO3 Imprinted Locus-Containing miRNAs

**Figure 2:**
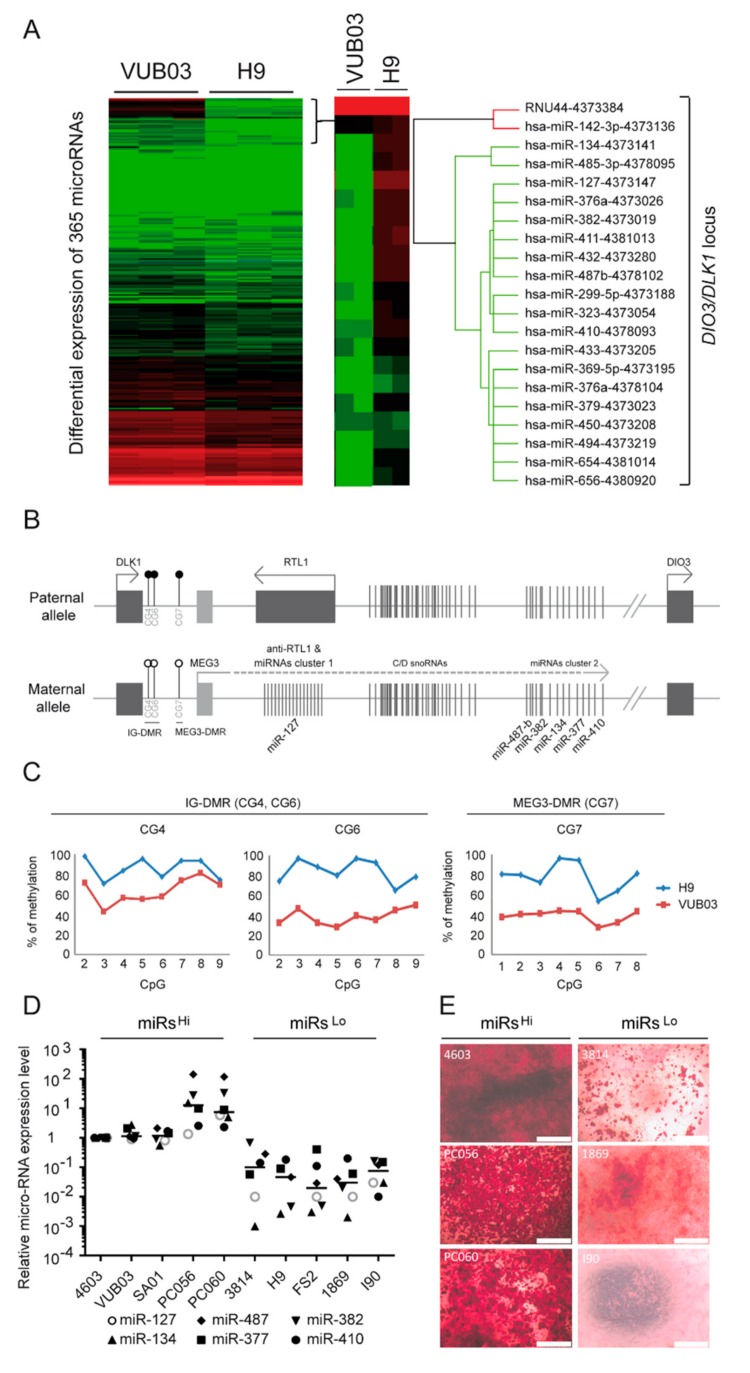
Silencing of microRNAs from the *DLK1/DIO3* locus in mesodermal progenitor cells with altered osteogenic differentiation potential. (**A**) Screening of microRNAs differentially expressed in VUB03 (high osteogenic potential) and H9_MPCs (low osteogenic potential) by quantitative RT-PCR Taqman arrays. Data were obtained from 3 independent experiments. (**B**) Schematic representation of genes and microRNA organization within the imprinted *DLK1/DIO3* locus. (**C**) Methylation status analysis of IG-DMR (CG4, CG6) and *MEG3*-DMR (CG7) CpG islands present in the regulatory sequences of the *DLK1/DIO3* locus from VUB03 and H9_MPCs. (**D**) Profiling of 6 microRNAs located along the *DLK1/DIO3* locus in MPCs derived from ten hPSC lines by RT-qPCR. Each microRNA is represented by a particular symbol. (**E**) Alizarin red staining of MPCs derived from the 4603, PC056, PC060, 3814, 1869, and I90 hPSC lines, cultured for twenty days in osteogenic differentiation medium. Data obtained in 3 independent experiments in duplicate.

Different microRNAs (miRNAs) have been demonstrated to play a critical role during the osteogenic differentiation process [26,27,28]. We thus sought to determine whether the differential osteogenic potential observed between the different hPSC_MPCs could be associated with a different miRNA expression profile. TaqMan arrays were therefore used to screen the expression profile of 365 miRNAs in VUB03 versus H9 hESC_MPCs. This analysis highlighted the downregulation of 53 microRNAs in H9 hESC-MPCs, which were characterized by a poor osteogenic capacity compared to VUB03 hESCs_MPCs, which in contrast present a robust osteogenic potential (Figure 2A). Interestingly, 31 of them belong to a cluster of microRNAs located in the *DLK1/DIO3* locus submitted to parental imprinting on chromosome 14 (Figure 2B and Figure S2) [29,30,31]. MicroRNAs from this locus were only expressed from the maternal allele due to the DNA methylation patterns of two differentially methylated regions (DMRs) suppressing the transcription of paternal origin: intergenic differentially methylated region (IG-DMR) and *MEG3*-DMR [32,33]. Since the downregulation of microRNAs from this locus has previously been associated with a defect of parental imprinting [33], we investigated the methylation status of the two DMRs in VUB03 and H9 hESC_MPCs. Pyrosequencing experiments revealed that methylation level at both IG-DMR and the *MEG3*-DMR reached 90% in H9 hESC-MPCs, correlating with the methylation of alleles of both paternal and maternal origin and the silencing of the miRNA cluster (Figure 2C). In contrast, the percentage of DMR methylation observed in VUB03 hESC_MPCs averaged 50%, as expected for the expression of this microRNA cluster by the maternal allele only. TaqMan array and pyrosequencing analysis confirmed the silencing of these clustered miRNAs and the hypermethylation status of the *DLK1/DIO3* DMRs detected in undifferentiated VUB03 and H9 hESC lines (Figure S2).

To evaluate the relationship between osteogenic potential and miRNAs from the *DLK1/DIO3* locus, the expressions of six microRNAs located all along the locus (Figure 2B) were analyzed in the six previously studied hPSC-derived MPCs (including VUB03 and H9) and four additional hiPSC-derived MPCs (PC056, PC060, 1869, and I90) (Figure S2). This analysis revealed two groups of cells behaving differentially in terms of the expression of these miRNAs. One group presented a high expression level of the six different *DLK1/DIO3* locus-containing miRNAs (named miR^hi^), as observed in VUB03 hESC-MPCs, while the second group harbored a lower expression of these miRNAs (miR^lo^), similar to H9 hESC-MPCs (Figure 2D). Osteogenic differentiation of these different lines confirmed that only the miR^hi^ lines had an efficient capacity to give rise to osteoblasts, as determined by mineralization assays (Figure 2E). Overall, these results indicate a correlation between the ability of 10 different hPSC-derived MPCs to differentiate towards the osteogenic lineage and the expression profile of miRNAs from the *DLK1/DIO3* locus.

### 3.3. miRNAs from the DLK1/DIO3 Locus Impact the Expression of Receptors from the Transforming Growth Factor-β (TGFβ) Superfamily Controlling Osteogenesis

**Figure 3:**
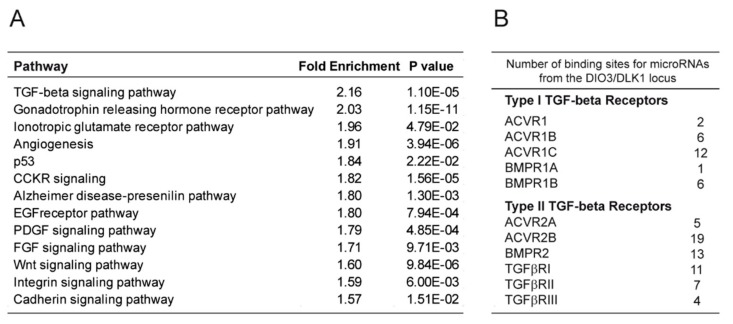

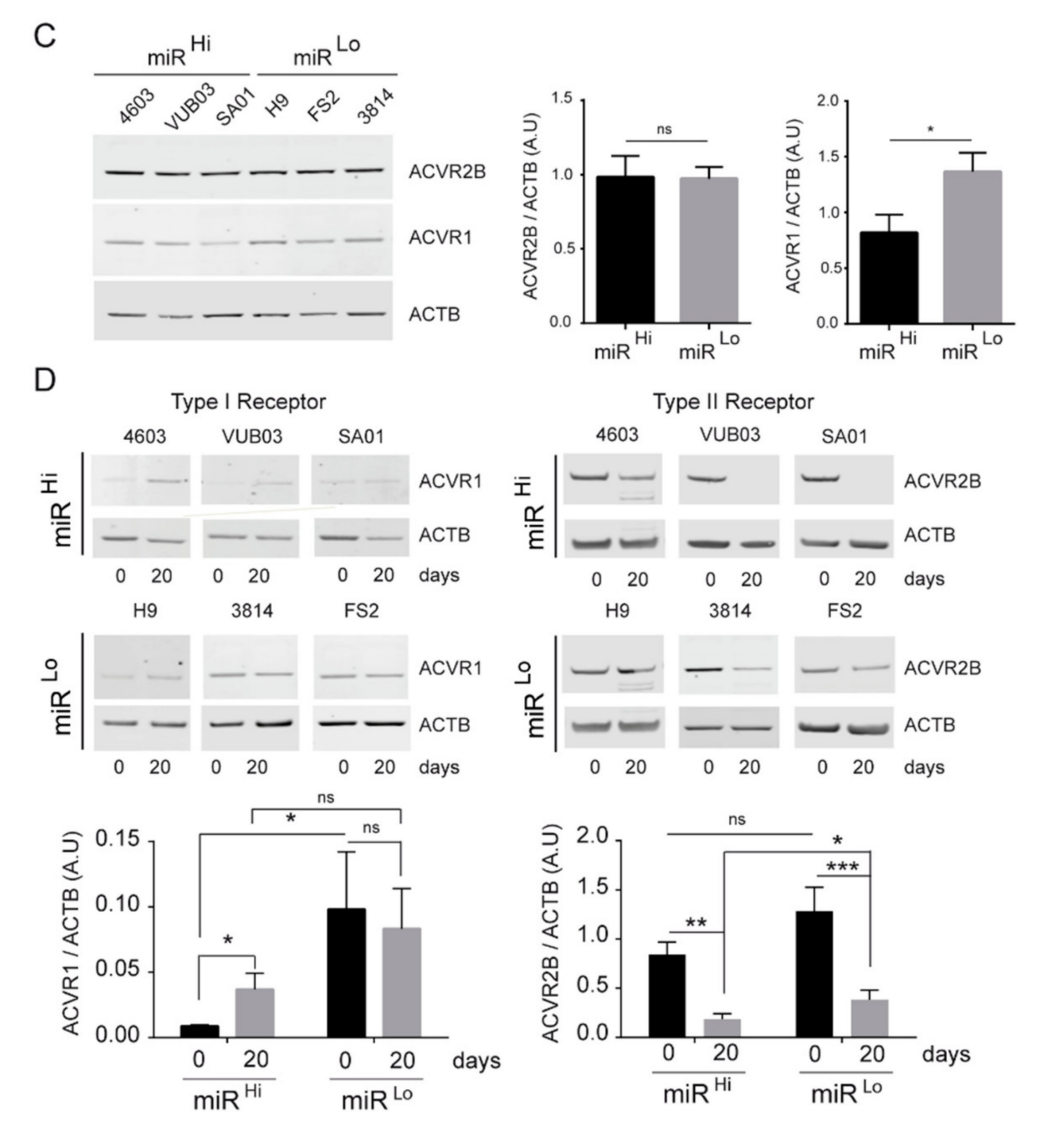
MicroRNAs from the *DLK1/DIO3* locus targets the expression of receptors from the transforming growth factor-β (TGFβ) superfamily. (**A**) Panther bioinformatic analysis of signaling pathways enriched among the targets of the 52 microRNAs from the *DLK1/DIO3* locus. (**B**) The number of binding sites predicted for microRNAs from the *DLK1/DIO3* locus within the 3′ untranslated sequence of genes encoding receptors of the TGFβ family (**C–D**) Comparison of ACVR1 and ACVR2B expressions in three MPCs^Lo^ and three MPCs^Hi^ cell lines on day 0 and day 20 of osteogenic differentiation by Western blot analysis. A.U: arbitrary units. Data were obtained from more than 2 independent experiments in triplicate and are represented as the mean ± SEM. Statistical analysis was based on a Mann–Whitney Test; * *p* < 0.05, ** *p* < 0.01, *** *p* < 0.001, ns: not significant.

To determine the mechanisms by which the miRNAs can influence the cell fate of human pluripotent stem cells toward the osteogenic lineage, we identified the putative targets of each of the 52 microRNAs from this locus using Targetscan [34] and investigated the molecular pathways enriched within this gene list with PANTHER software tools [35]. Interestingly, the TGFβ signaling pathway, known to be involved in the osteogenic specification process, appeared as the main pathway targeted by the miRNAs from the *DLK1/DIO3* locus (Figure 3A and Figure S3). In this pathway, we focused on *ACVR2B* and its coreceptor *ACVR1* (*ALK2*), both described as negative regulators of osteogenesis. Interestingly, these two genes displayed 19 and 2, respectively, predicted binding sites for microRNAs from the *DLK1/DIO3* locus within their 3’ untranslated region (UTR) (Figure 3B and Figure S3). We first measured the expression level of these receptors by Western blot analysis in miR^lo^ and miR^hi^ hPSC-MPCs and in derived osteogenic cells. We observed that ACVR1 expression was downregulated in miR^hi^ hPSC-MPCs compared to miR^lo^ hPSC-MPCs (Figure 3C). While its expression increased during differentiation in miR^hi^ hPSC-MPCs, its level of expression was stable in miR^lo^ hPSC-MPCs (Figure 3D). In contrast, ACVR2B was not differentially expressed between miR^lo^ and miR^hi^ hPSC-MPCs (Figure 3C). However, its downregulation during differentiation was statistically more efficient in miR^hi^ hPSC osteogenic derivatives in comparison with miR^lo^ osteogenic derivatives (Figure 3D). These results suggest that the expression level of miRNAs from the *DLK1/DIO3* locus influences the expression profile of members from the TGFβ superfamily receptors, such as ACVR1 and ACVR2B, throughout the hPSC-MPC osteogenic differentiation process.

### 3.4. Functional Impact of miRNAs from the DLK1/DIO3 Locus on ACVR2B Receptor Signal Transduction

**Figure 4:**
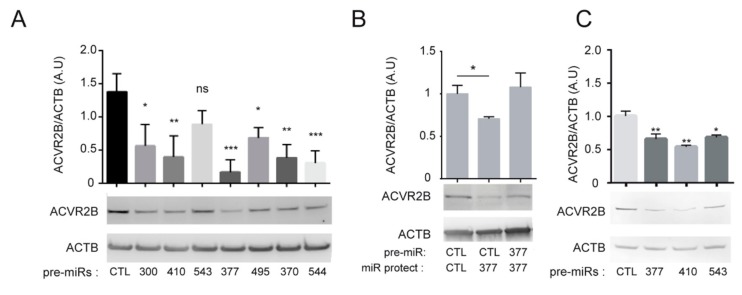

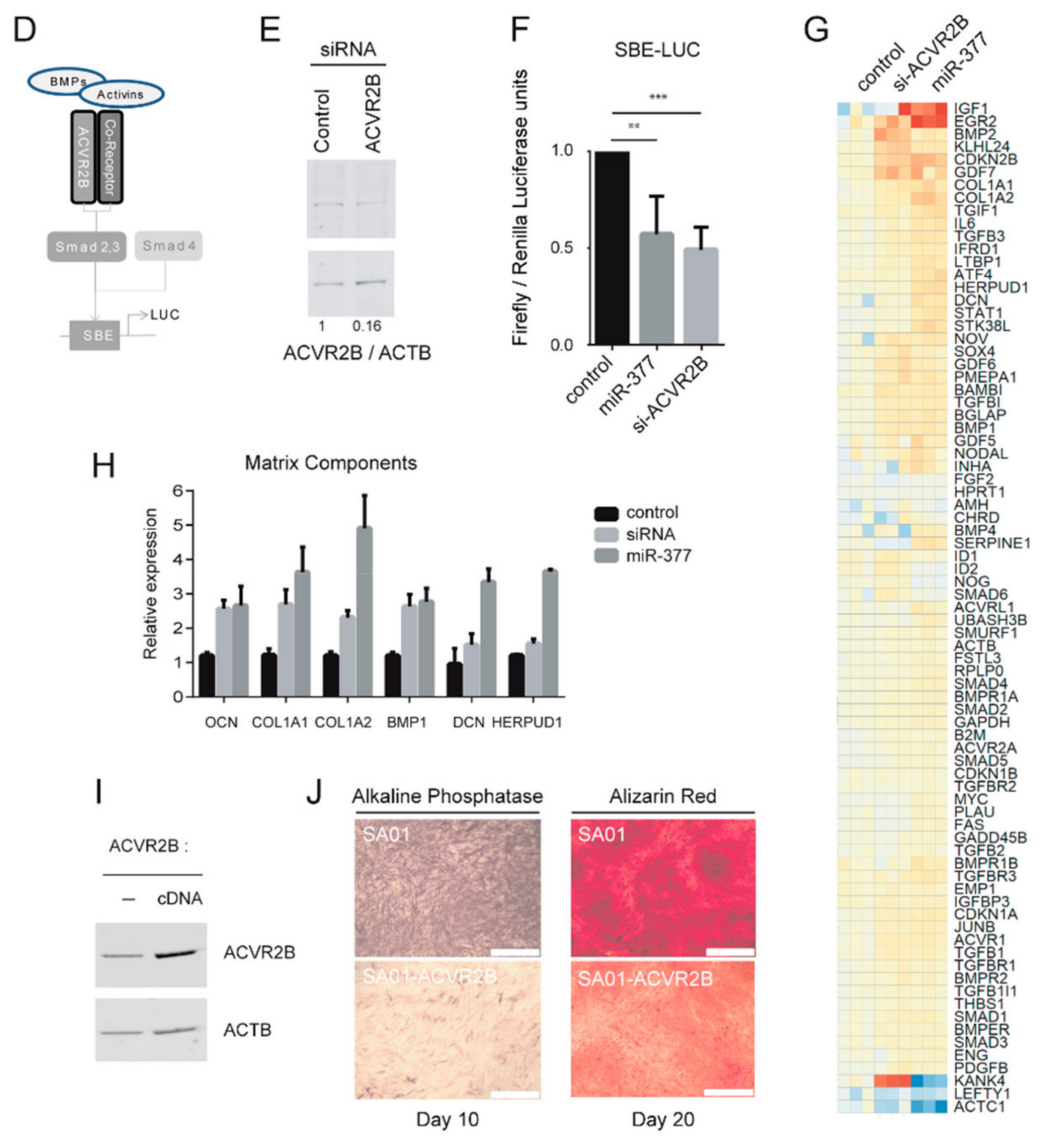
Modulation of ACVR2B expression by miR-377-3p modifies receptor signaling and alters the osteogenic potential of hPSC-derived MPCs. (**A**) Western blot analysis of ACVR2B expression in SA01_MPCs transfected for 72 h with 7 pre-microRNAs. Data collected from 3 independent experiments are represented as the mean ± SEM and were statistically analyzed with one-way ANOVA followed by Dunnett”s multiple comparison test; * *p* < 0.05, ** *p* < 0.01, *** *p* < 0.001, ns: not significant. (**B**) Western blot analysis of ACVR2B expression in SA01_MPCs cotransfected for 72 h with microRNA-377-3p together with an miRProtect sequence used to mask the microRNA-377-3p binding site in *ACVR2B* 3’UTR. Data from 2 independent experiments are represented as the mean ± SEM and were analyzed with an impaired *t*-test; *p < 0.05. (**C**) Western blot analysis of ACVR2B expression in HEK293T cells transfected for 72 h with 3 pre-microRNAs. Data collected from 3 independent experiments are represented as the mean ± SEM and were statistically analyzed with one-way ANOVA followed by Dunnett”s multiple comparison test; * *p* < 0.05, ** *p* < 0.01, *** *p* < 0.001, ns: not significant. (**D**) Schematic representation of SMAD activation downstream of TGFβ superfamily receptor activation. (**E**) Western blot analysis of ACVR2B expression in HEK (Human Embryonic Kidney cells) transfected for 72 h with an siRNA control or specific to *ACVR2B*. (**F**) Luciferase activity detection of reporters carrying SMAD2/3 responsive elements cotransfected with an siRNA control, siRNA specific to *ACVR2B,* or a pre-miR-377 for 24 h in HEK cells. Data from 3 independent experiments in triplicates are represented as the mean ± SD. Statistical analysis was based on one-Way ANOVA followed by a Dunnet’s multiple comparison test; ** *p* < 0.01, *** *p* < 0.001. (**G**,**H**) Screening of differentially expressed genes from the TGFβ pathway using RT-QPCR Taqman arrays in SA01_MPCs 72 h after transfection with an siRNA control or specific to *ACVR2B*. (**I**) Western blot analysis of ACVR2B expression in SA01_MPCs stably transfected with the pAAVS1-P-CAG-*ACVR2B* vector. (**J**) Alkaline phosphatase and alizarin red staining of SA01_MPC control or stably transfected with the pAAVS1-P-CAG-*ACVR2B* vector ten and twenty days after osteogenic differentiation. Data have been observed in 3 independent experiments (Scale bar: 500 µM).

To explore the relationship between miRNAs from the *DLK1/DIO3* locus and TGFβ superfamily receptors, we focused on ACVR2B expression, as this receptor displays 19 predicted binding sites for microRNAs from the *DLK1/DIO3* locus in its 3′ untranslated region (UTR) (Figure S3) and is downregulated in osteogenic derivatives (Figure 3D). For this purpose, seven miRNA mimics targeting its 3’ UTR sequence were chemically synthesized and overexpressed by transient transfection in SA01_MPCs. Western blot analysis confirmed the downregulation of ACVR2B in response to miRNAs-300, 410-3p, 495-3p, 370-3p, and 544a, and more markedly, 377-3p (Figure 4A). The possibility of an indirect effect of miRNA-377-3p overexpression on ACVR2B downregulation was excluded by the cotransfection of an oligonucleotide that masked the target sequence for miRNA-377-3p on the 3′ UTR of ACVR2B and abolished the downregulation of ACVR2B (Figure 4B). The impact of miRNAs mimic-377-3p and 410 on ACVR2B expression was also confirmed in HEK293T cells by Western blot, indicating that this regulation occurs independently of the cellular context (Figure 4C). To extend these results to the functional level, we next studied the impact of miRNA-377-3p on the ACVR2B signaling pathway. It has already been described that ACVR2B signal transduction is mediated through the activation of transcription factors SMAD2/3 [36] (Figure 4D). To test the effect of miRNA-377-3p on this signaling pathway, the miRNA-377-3p mimic or the siRNA targeting *ACVR2B* (Figure 4E) was cotransfected with an SMAD-binding element luciferase reporter in HEK293T cells. The overexpression of miRNA-377-3p induced a decreased luciferase expression, equivalent to that mediated by ACVR2B downregulation by RNA interference, suggesting a functional impact of miRNA-377-3p on ACVR2B signaling (Figure 4F). This result was also confirmed by analyzing the molecular targets of the SMAD signaling pathway using a TGFβ/BMP signaling pathway PCR array. The transient transfection of the miRNA-377-3p mimic or siRNA-ACVR2B in SA01_MPCs for 72 h led to an increased expression of a set of genes involved in the induction of the osteogenic differentiation, such as *BMP2*, *GDF7,* and *IGF1,* or associated with markers of differentiation (*OCN*, *COL1A1*, *COL1A2*, *BMP1*, *DCN,* and *HERPUD1*) (Figure 4G,H and Figure S4). Conversely, we tested the effect of *ACVR2B* overexpression on the osteogenic differentiation of miR^hi^ hPSC-MPCs. We stably transfected the miR^hi^ SA01-MPCs with a plasmid encoding *ACVR2B* and observed that stable ACVR2B overexpression in the SA01 hESC line did not prevent the derivation of MPCs (Figure 4I, Figure S2). However, the ability of these cells to differentiate towards the osteogenic lineage was greatly impaired, as assessed by alkaline phosphatase and alizarin red staining on days ten and twenty of the differentiation (Figure 4J). Together, these results demonstrate the functional impact of miRNAs from the *DLK1/DIO3* locus on ACVR2B signal transduction and the osteogenic differentiation of hPSC-MPCs.

## 4. Discussion

The main finding of this study was the identification of molecular mechanisms that underlie osteogenic variability between different human pluripotent stem cells lines, opening the possibility of an anticipating screening of cell lines of interest. These mechanisms involve the imprinted *DLK1/DIO3* locus, the epigenetic control of which appears to be highly predictive of the osteogenic potential of human pluripotent stem cells lines. The functional impact of differentially expressed miRNAs comes, at least in part, from the signaling pathways triggered by the activation of the activin receptor 2B.

The key practical application of our findings is the use of miRNA analysis from the *DLK1/DIO3* imprinted locus to predict the osteogenic capacity of human pluripotent stem cells. The development of protocols to specify hPSC lines into functional osteogenic cells has led the skeletal biology field to harness the potential of hPS cells as a critical model system for understanding diseases of abnormal skeletal formation and bone regeneration. Mature and phenotypically stable in vivo bone substitutes have successfully been engineered from hESC and hiPSC [37,38]. Different studies have also demonstrated the potential of hPSC to phenocopy osteogenic defects associated with genetic disorders such as progressive fibrodysplasia ossificans or Marfan syndrome [39,40]. Nonetheless, these different applications of hPSCs are hindered by the differentiation inconsistency of individual hPSC lines and the fact that the molecular mechanism underlying the interline variability has remained elusive [41]. Based on the analysis of a dozen hPSC lines that replicated the observed variability in osteogenic capacity, we demonstrated that a reduced expression of miRNAs belonging to the imprinted *DLK1/DIO3* locus was associated with a low osteogenic yield of hPSC lines. Interestingly, the loss of miRNA expression from the *DLK1/DIO3* locus was previously shown to correlate with reduced neural differentiation in hESC lines [10]. In addition, mouse iPSCs with an aberrantly hypermethylation of the *Dlk1/Dio3* locus and consequently, repressed miRNAs, have been reported to not achieve developmental potential for generating full-term mice in a tetraploid complementation assay [42,43]. Together, these results suggest that the expression of miRNAs from the *DLK1/DIO3* locus may be used more broadly as a marker of in vitro capacity of differentiation of hPSCs.

It is currently unclear whether these variations in gene expression and capacity of differentiation reflect a distinct developmental origin of the lines or can be influenced by culture conditions. Changes have been reported in imprinting in mouse and human PSC lines as a result of either prolonged culture or the reprogramming process [43,44,45,46,47], whereas others have found imprinting to be relatively stable [46,48]. These findings emphasize the importance of developing methods to minimize the differences of hPSC capacities. The purification of proliferative progenitors during the process of differentiation may be a way to attenuate the variability between hPSCs. Such an approach has already been applied to different types of hPSC-derived progenitors, but its benefit still remains to be proven [49,50,51]. Variability in differentiation propensity is likely to occur at different stages of differentiation and thus, could require the purification of several intermediates [52]. An alternative approach to attenuate this is to acquire a better understanding of the molecular mechanisms directing hPSC cell fate decisions and in particular, identify how the genetic and epigenetic background can influence these mechanisms.

At the mechanistic level, our results point to a differential response to TGFβ signaling among cell lines that is in part related to the varying level of different miRNAs from the *DLK1/DIO3* locus. By analyzing the putative targets of these microRNAs, we identified the ACVR2B activating receptor as a functional target of at least six different microRNAs of the cluster. Within the TGFβ superfamily, the type 2 receptor ACVR2B binds with the highest affinity to activin A and BMP3, two negative regulators of osteogenic differentiation [53,54]. Illustrating the importance of this receptor in bone formation, its inactivation has even been considered as a promising therapeutic target for diseases characterized by loss of muscle and bone mass. Preclinical and clinical studies have demonstrated that injection of a recombinant protein, including the extracellular domain of ACVR2B receptor fused to the Fc region of immunoglobulin, led to increased bone volume [55,56,57,58,59].

Different studies have already shown that the expression of ACRV2B receptor can be modulated by miRNAs [60,61,62]. However, the demonstration that microRNA from the *DKL1/DIO3* locus controls the ACVR2B receptor contributes to highlight that this imprinted locus is a master regulator of osteogenesis. Indeed, the BMP pathway is also controlled by the maternally expressed gene *MEG3* that induces osteogenesis through the induction of BMP4. Conversely, the paternally expressed *DLK1* that belongs to the Notch/Serrate/Delta family is a negative regulator of osteogenesis [63,64]. Concordant with these data, in mouse embryos with uniparental disomy for chromosome 12, in which both copies of that chromosome containing the ortholog *DLK1/DIO3* locus are paternally derived, hypo-ossification of mesoderm-derived bones was observed [65,66]. Interestingly, ascorbic acid has been shown to maintain the miRNA expression of the *Dlk1/Dio3* locus in mouse and human embryonic stem cells. In further studies, analyzing the impact of ascorbic acid on the potential of osteogenic differentiation from different hPSC lines may have a great impact on the improvement of the differentiation propensity of hPSCs.

## 5. Conclusions

Our study stresses the importance of identifying molecular markers in the pluripotent state that are predictive of the differentiation behavior of hESCs and hiPSCs. The identification of such markers would help in the selection of the most suitable cell lines for specific applications.

## Supplementary Materials

The following are available online at https://www.mdpi.com/2073-4409/8/12/1523/s1, Figure S1: Characterization of MPCs derived from human pluripotent stem cells, Figure S2: Identification of the differentially expressed miRNAs between hESC lines harboring a variable osteogenic capacity, Figure S3: Table describing the number of miRNA binding sites from the *DLK1/DIO3* locus predicted in the 3′ UTR of receptors of the TGFβ pathway, Figure S4: Detail of genes modulation detected using the TGFβ/BMP signaling pathway plus PCR array card, Table S1: Primer sequences and assays used for genes and microRNAs quantifying.
