# Supplementary material for: Expression of miRNAs from the Imprinted DLK1/DIO3 Locus Signals the Osteogenic Potential of Human Pluripotent Stem Cells
Source: Cells. 2019 Nov 26;8(12):1523. doi: 10.3390/cells8121523 (PMC6953034; doi:10.3390/cells8121523)
Supplement: Supplementary file 1 [file cells-08-01523-s001.pdf]

A

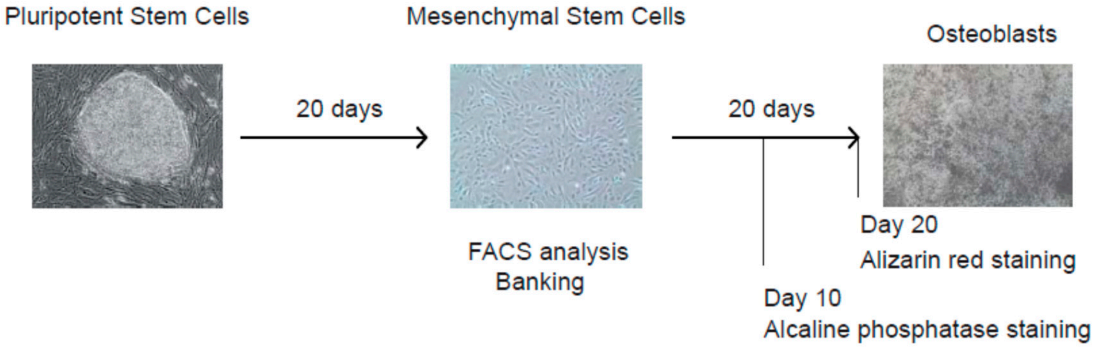

B

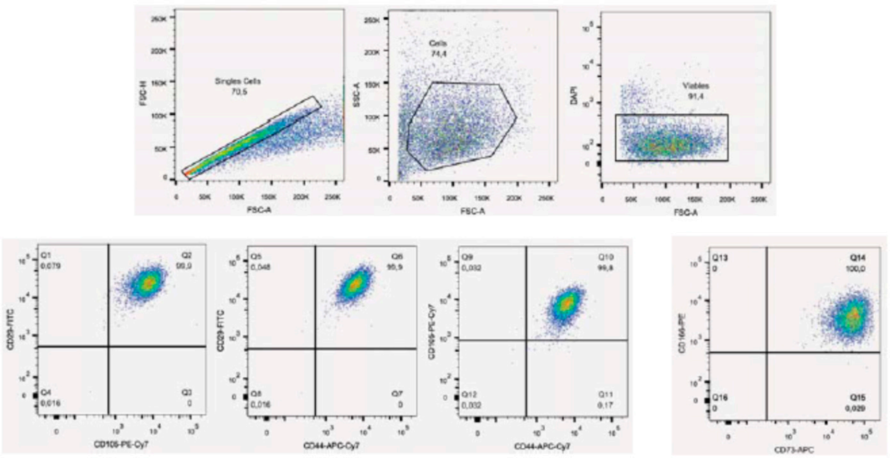

C

Markers (%)	MSC lines					
	H9	SA01	VUB03	3814	FS2	4603
CD29/CD105	99,9	96	100	94,5	96,2	99,8
CD29/CD44	99,9	99,6	97,1	96,3	98,2	97,6
CD105/CD44	99,8	95,8	97,8	94,6	95,8	97,7
CD166/CD73	100	99,3	99,1	90,8	94,9	99,2

Figure S1. Characterization of MPCs derived from human pluripotent stem cells. (**A**) Schematic representation of the protocol used for osteogenic differentiation of hPSCs_MPCs. (**B**) Gating strategy and staining representation of MPC surface antigen marker expression analyzed by flow cytometry. (**C**) Flow cytometry analysis of mesenchymal markers CD73, CD29, CD105, CD44, CD166 in MPCs derived from three human embryonic stem cell lines (H9, SA01 and VUB03) and three induced pluripotent stem cell lines (3814, FS2 and 4603).

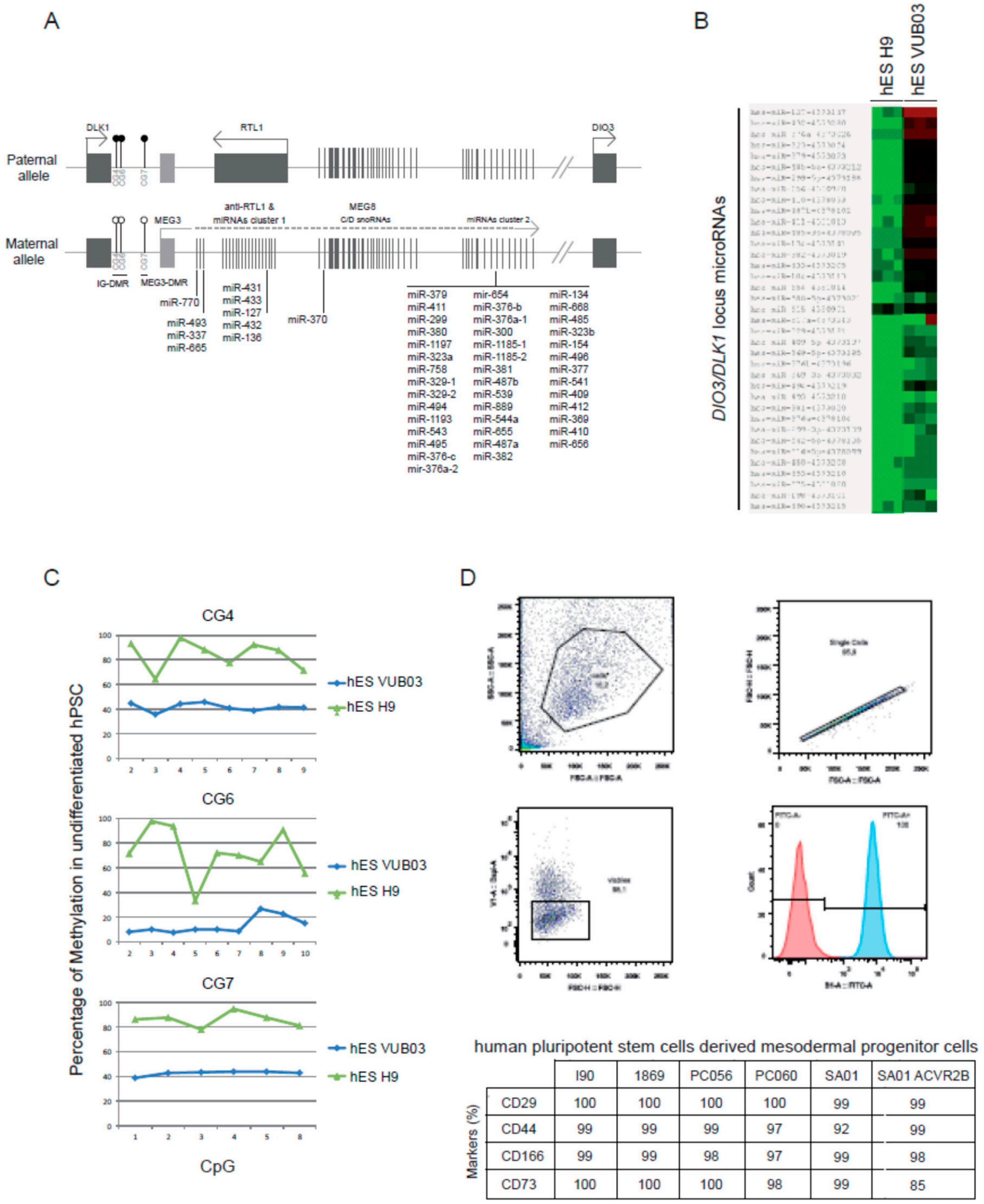

	I90	1869	PC056	PC060	SA01	SA01 ACVR2B
CD29	100	100	100	100	99	99
CD44	99	99	99	97	92	99
CD166	99	99	98	97	99	98
CD73	100	100	100	98	99	85

Figure S2. Identification of the differentially expressed miRNAs between hESC lines harboring a variable osteogenic capacity. (**A**) Schematic representation of gene and microRNA organization within the imprinted *DLK1/DIO3* locus. (**B**) Screening of microRNAs differentially expressed in VUB03 and H9 human embryonic stem cell lines by quantitative RT-PCR Taqman Arrays. Data were obtained from 3 independent samples. (**C**) Methylation status analysis of IG-DMR (CG4, CG6) and MEG3-DMR (CG7) CpG islands present in the regulatory sequences of the *DLK1/DIO3* locus from VUB03 and H9 human embryonic stem cell lines. (**D**) Flow cytometry analysis of mesenchymal markers CD29, CD44, CD166 and CD73 in MPCs derived from the four induced pluripotent stem cell lines i90, 1869, PC056 and PC060, the embryonic stem cell line SA01 and the *ACVR2B*-overexpressing SA01 cell line.

	Type 1 TGFb receptors family					Type 2 TGFb receptors family		
	ACVR1	ACVR1B	ACVR1C	BMPR1A	BMPR1B	ACVR2A	ACVR2B	BMPR2
DIO3/DLK1 microRNA		miR-770		miR-136		miR-770	miR-770	miR-136
							miR-370-3p	
		miR-337						
							miR-323a-3p	
			miR-432				miR-411	miR-329-3p
			miR-494_3p			miR-380	miR-380	miR-494_3p
			miR-543			miR-543	miR-543	
		miR-496			miR-495-3p		miR-495-3p	
								miR-376a-3p
			miR-654-3p				miR-654-3p	
								miR-376b
								miR-376a
			miR-487b		miR-300		miR-300	miR-300
		miR-1185					miR-1185	
	miR-381				miR-381		miR-381	miR-381
							miR-487b	
			miR-889-3p		miR-889-3p	miR-889		miR-889-3p
		miR-889	miR-889-3p		miR-889-3p			miR-889-3p
	miR-544						miR-544a-3p	miR-544a-3p
			miR-382					miR-655
							miR-323b	
			miR-485-3p					
							miR-154	
							miR-377-3p	
			miR-541			miR-409-5p	miR-409	
			miR-409-3p					
							miR-369-3p	miR-369-3p
			miR-410-3p				miR-410-3p	
		miR-656-3p	miR-656-3p		miR-656-3p			

Figure S3. Table describing the number of miRNA binding sites from the *DLK1*/*DIO3* locus predicted in the 3′UTR of receptors of the TGFβ Pathway.

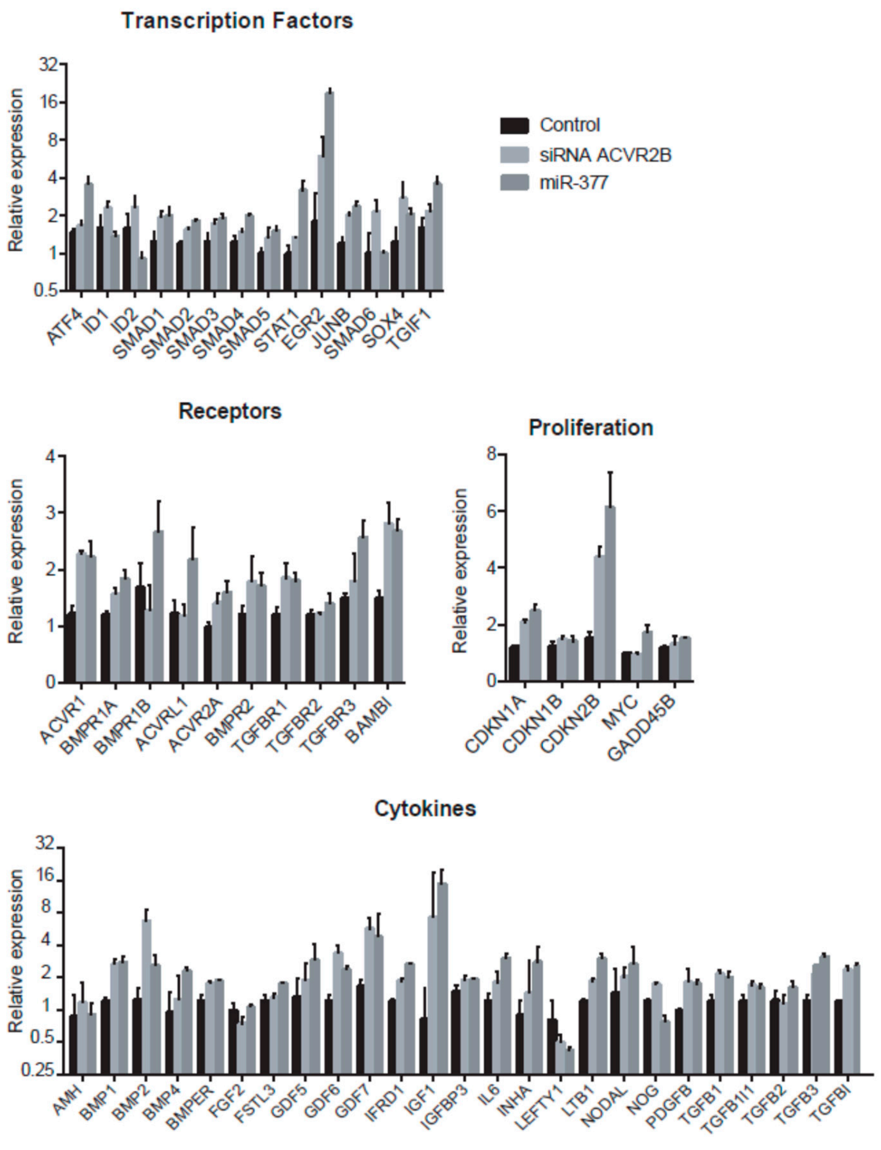

Figure S4. Detail of genes modulation detected using the TGFβ/BMP Signaling Pathway Plus PCR Array card. Analysis of gene regulation quantified by RT-QPCR in SA01-MPCS transfected with an siRNA control, siRNA specific for *ACVR2B* or a pre-miR-377-3p. Genes were classified into the following categories: Transcription factors, Receptors, Proliferation and Cytokines. Data in triplicates are represented as the mean ± SEM.

Table S1. Primer sequences and assays used for genes and microRNAs quantifying.

Gene	Primer	Sequence
RUNX2	RunX2 Fw	5′-ttacttacaccccgccagtc-3′
	RunX2 Rev	5′-tatggagtgctgctggtctg-3′
ALPL	ALPL Fw	5′-ccacgtcttcacatttggtg-3′
	ALPL Rev	5′-agactgcgcctggtagttgt-3′
OCN	OCN Fw	5′-gctgagtcctgagcagcag-3′
	OCN Rev	5′-ccaatagggcgaggagtgtg-3′
18S	18S Fw	5′-gaggatgaggtggaacgtgt-3′
	18S Rev	5′-tcttcagtcgctccaggtct-3′

**miScript miRNA Mimic**	**Cat. No.**
Syn-hsa-miR-300	MSY0004903
Syn-hsa-miR-410-3p	MSY0002171
Syn-hsa-miR-543	MSY0004954
Syn-hsa-miR-377-3p	MSY0000730
Syn-hsa-miR-495-3p	MSY0002817
Syn-hsa-miR-370	MSY0000722
Syn-hsa-miR-544a	MSY0003164

**miScript Primer Assay**	**Cat. No.**
Hs_miR-127_1	MS00003437
Hs_miR-377_1	MS00004095
Hs_miR-487b_1	MS00004298
Hs_miR-382_2	MS00031836
Hs_miR-134_2	MS00031437
Hs_RNU6-21	MS00033740
